# Supplementary material for: miRNA Expression Analysis of the Hippocampus in a Vervet Monkey Model of Fetal Alcohol Spectrum Disorder Reveals a Potential Role in Global mRNA Downregulation
Source: Brain Sci. 2023 Jun 9;13(6):934. doi: 10.3390/brainsci13060934 (PMC10296739; doi:10.3390/brainsci13060934)
Supplement: Supplementary file 1 [file brainsci-13-00934-s001.zip › brainsci-2433835-supplementary.pdf]

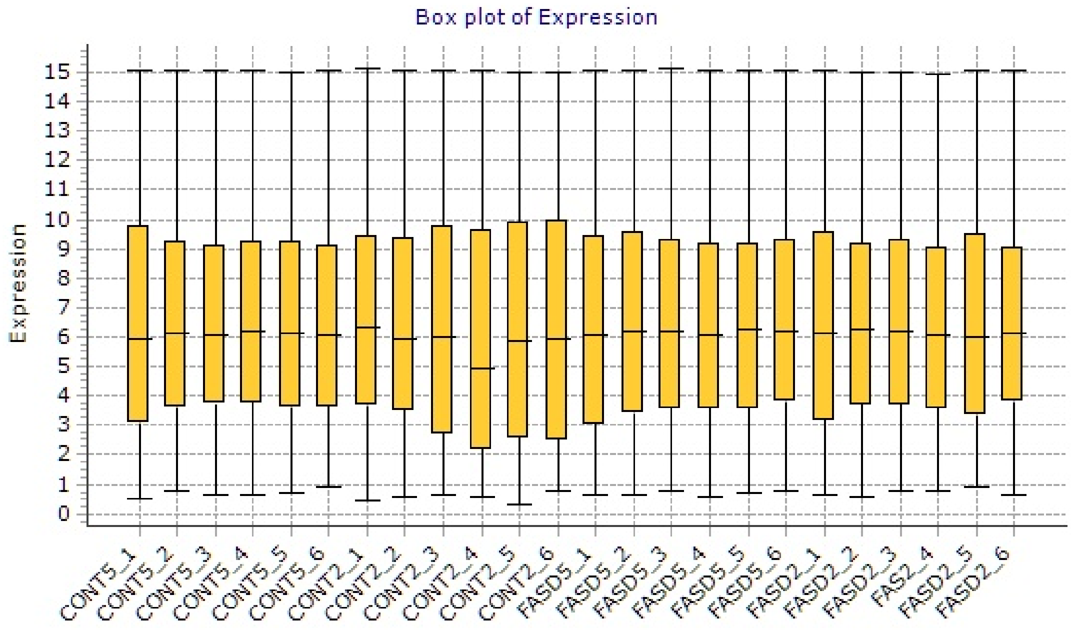

Supplemental Figure S1: Box plot diagram of 24 miRNA genechip 3.0 post probe filtering using only the 619 miRNA probes interrogated within this study. CONT2_4 revealed a lower mean expression level and lower quartile boundary and was therefore excluded from further analysis.

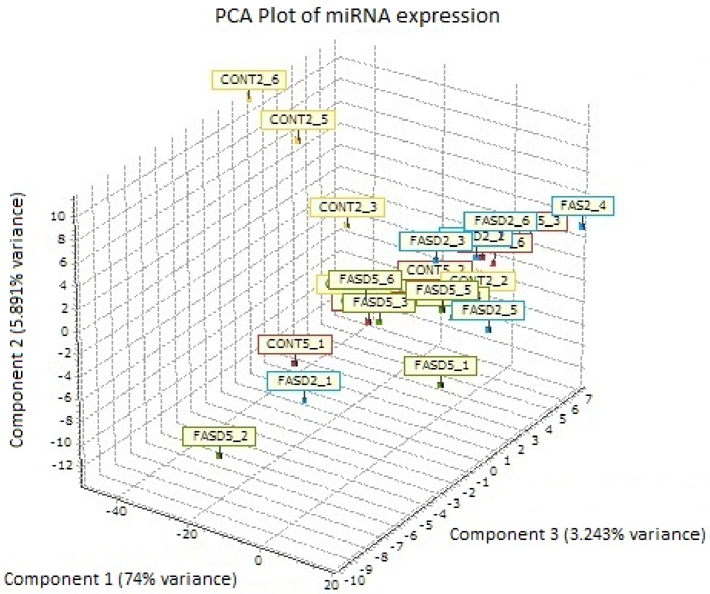

Supplemental Figure S2: Principle component analysis of 23 remaining miRNA 3.0 genechip arrays revealing the unsupervised organization based on expression levels of the 619 miRNAs interrogated within this study

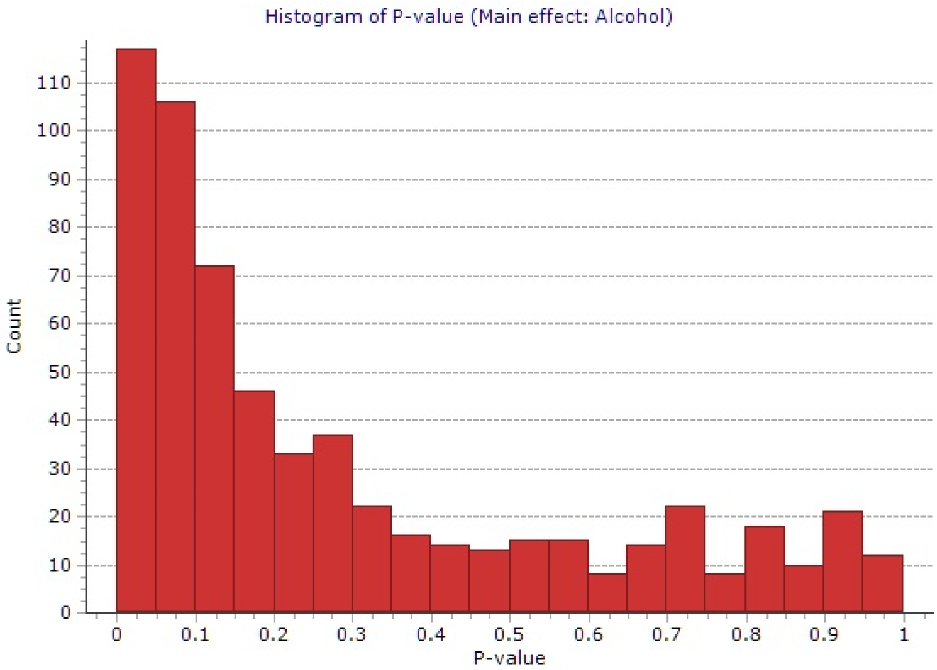

Supplemental Figure S3: Histogram of *p*-values using Alcohol as a main effect for the 619 miRNAs interrogated in the miRNA 3.0 genechip microarray in the offspring of alcohol preferring vervet dams vs sucrose matched controls.

Differentially Expressed miRNAs using Alcohol as Main Effect

miRNA	*p*-value Alcohol	*q*-value	log2(Fold change)
miR-548a	0.0003	0.096	-1.19
miR-340-5p	0.0144	0.113	1.20
miR-505-5p	0.0150	0.113	1.55
miR-137-3p	0.0153	0.113	2.06
miR-21-5p	0.0163	0.113	1.45
miR-539-5p	0.0168	0.113	1.26
miR-182-5p	0.0169	0.113	1.34
miR-186-5p	0.0172	0.113	1.25
miR-18a-5p	0.0192	0.113	1.16
miR-9-5p	0.0195	0.113	1.25
miR-4487	0.0203	0.113	-1.42
miR-489-3p	0.0203	0.113	1.07
miR-363-3p	0.0214	0.113	1.47
miR-20a-5p	0.0259	0.113	1.06
miR-16-5p	0.0262	0.113	1.79
miR-218-5p	0.0278	0.113	1.33
miR-376c-3p	0.0299	0.113	1.55
miR-329-3p	0.0309	0.113	1.32
miR-24-1-5p	0.0339	0.113	1.29
miR-34b-5p	0.0343	0.113	1.39
miR-199a-3p	0.0418	0.113	1.51
miR-101-3p	0.0434	0.113	1.35
miR-204-5p	0.0440	0.113	1.35
miR-543	0.0465	0.113	1.40
miR-495-3p	0.0483	0.113	1.32
miR-4745-5p	0.0488	0.113	-1.02
miR-301a-3p	0.0499	0.113	1.45

Supplemental Table S1: List of miRNAs that revealed a *p*-value <0.05 and a log2 fold change greater than 1 or less than -1 when interrogating Alcohol as a main effect.

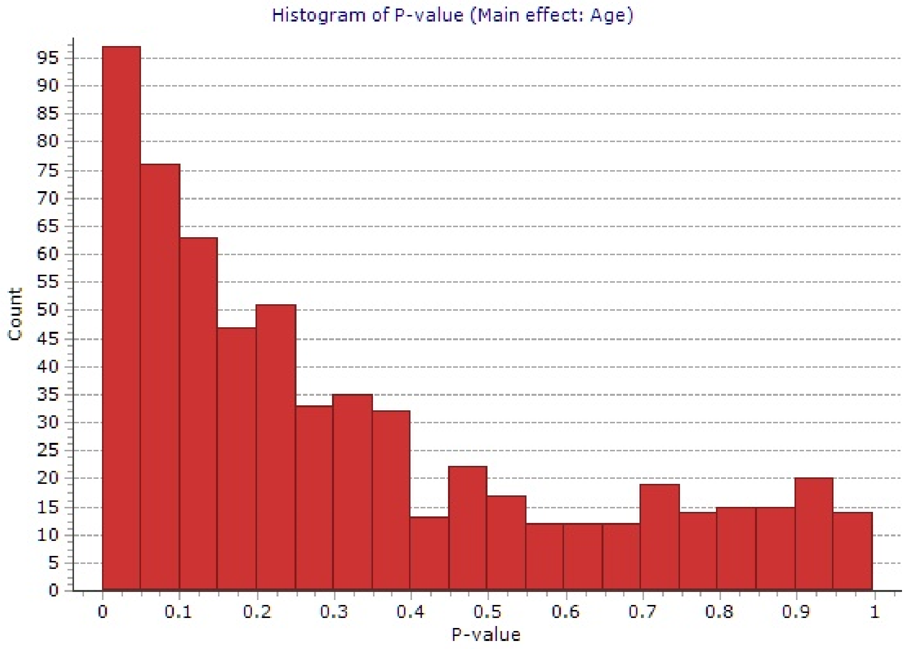

Supplemental Figure S4: Histogram of *p*-values using Age as a main effect for the remaining 619 miRNAs interrogated on the miRNA genechip 3.0 microarray contrasting gene expression at 5 months and 2 years old.

Differentially Expressed miRNAs using Age as a Main Effect

**miRNA**	***p*-value age**	***q*-value**	**log2(Fold change)**
miR-451a	0.0028	0.1203	-1.26
miR-2355-3p	0.0033	0.1203	-1.07
miR-9-5p	0.0065	0.1203	-1.59
miR-24-2-5p	0.0128	0.1273	-1.12
miR-1281	0.0223	0.1403	1.24
miR-505-5p	0.0242	0.1403	-1.48
miR-34b-5p	0.0267	0.1403	-1.54
miR-103a-2-5p	0.0268	0.1403	-1.11
miR-204-5p	0.0330	0.1403	-1.52
miR-376c-3p	0.0407	0.1403	-1.52
miR-539-5p	0.0418	0.1403	-1.09
miR-18a-5p	0.0431	0.1403	-1.03
miR-186	0.0461	0.1416	-1.07
miR-20a	0.0495	0.1458	-1.42

Supplemental Table S2: List of miRNAs which revealed a *p*-value <0.05 with a fold change greater than 1 or less than -1 with their corresponding *q*-values while using Age as a main effect.

Interaction Plots for 9 miRNAs with lowest Interaction *p*-values

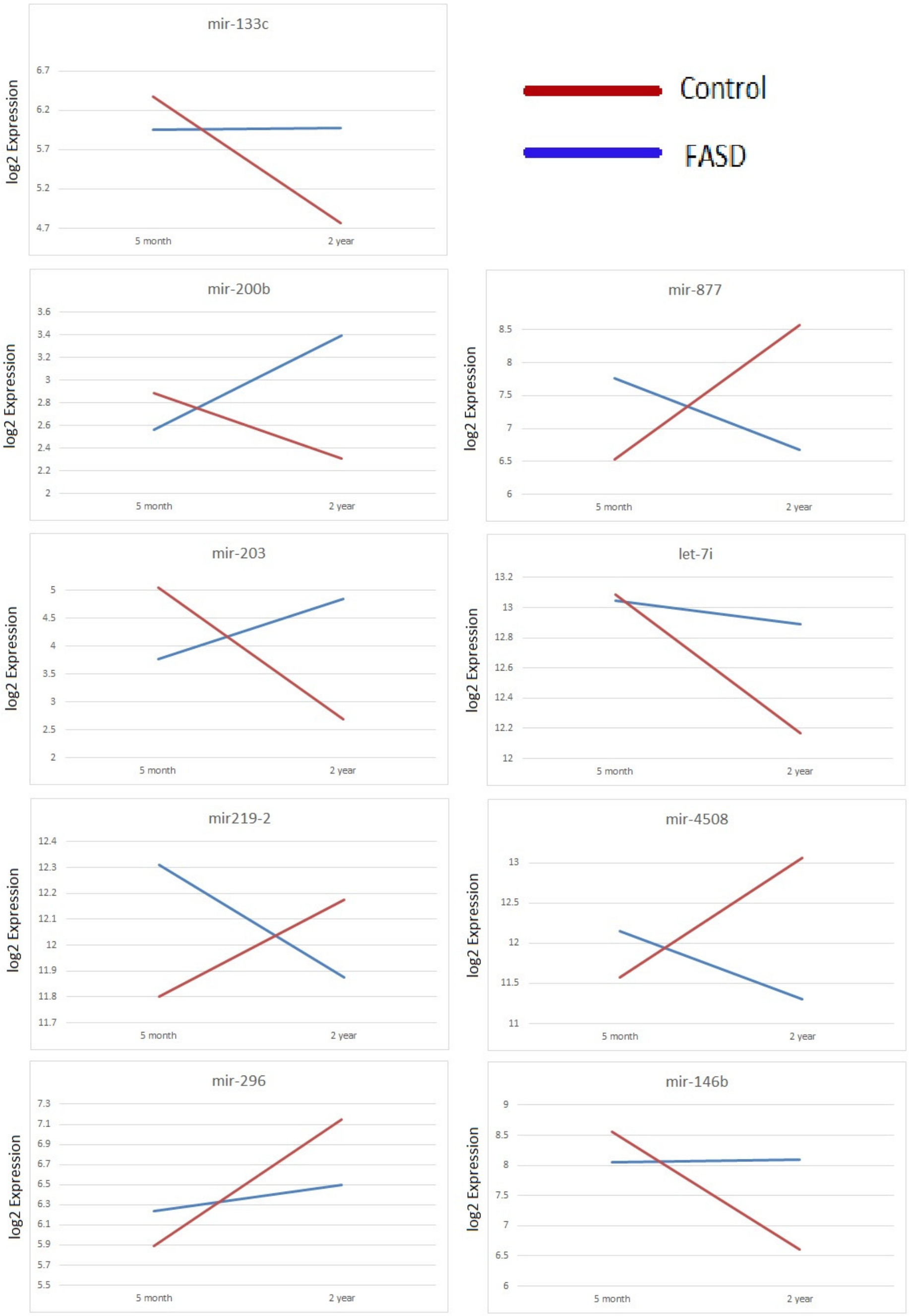

Supplemental Figure S5: Sample of 9 interaction plots for miRNAs that had the lowest *p*-values using Interaction as the main effect. The plots display the interaction type between

our two independent variables Age (5 months, 2 years) and Alcohol (FASD, Control).

Differentially Expressed miRNAs Derived from Interaction

miRNA	***p*-value interaction**	***q*-value**
miR-133a-3p	0.0013	0.02
miR-200b-3p	0.0019	0.02
miR-146b-5p	0.0023	0.02
miR-203a-3p	0.0024	0.02
miR-877-5p	0.0033	0.02
let-7i-5p	0.0034	0.02
miR-4508	0.0036	0.02
miR-219a-2-3p	0.0038	0.02
miR-296-3p	0.0038	0.02
miR-29a	0.0039	0.02
miR-4645-5p	0.0040	0.02
miR-4500	0.0043	0.02
miR-299-3p	0.0044	0.02
miR-3960	0.0052	0.02
miR-3064-5p	0.0053	0.02
miR-3668	0.0058	0.02
miR-564	0.0058	0.02
miR-3656	0.0058	0.02
miR-15a-5p	0.0062	0.02
miR-466	0.0063	0.02
miR-4510	0.0067	0.02
miR-638	0.0068	0.02
miR-3665	0.0068	0.02
miR-103a-2-5p	0.0069	0.02
miR-4734	0.0075	0.02
miR-143	0.0077	0.02
miR-1469	0.0077	0.02
miR-1908-5p	0.0078	0.02
let-7a-2-3p	0.0080	0.02
miR-1273c	0.0080	0.02

Supplemental Table S3: List of 31 miRNAs with the lowest *p*-values with their corresponding *q*-values when exploring the data using Interaction as the main effect.

**miRNA**	***p-value alcohol***	***q*-value**
miR-214-3p	0.9981395	0.4501469
miR-297	0.9974107	0.4501469
miR-93-5p	0.9966412	0.4501469
miR-3148	0.9943829	0.4501469
miR-181a-5p	0.9917749	0.4501469
miR-3174	0.9910066	0.4501469
miR-503-5p	0.9761876	0.4445561
miR-502-3p	0.963095	0.4393103
miR-1252-5p	0.9580473	0.4377231
miR-4314	0.9577089	0.4377231
miR-4764-5p	0.9568686	0.4377231
miR-548j-5p	0.9557681	0.4377231
miR-654-5p	0.9465216	0.4353069
miR-4253	0.9452107	0.4353069
miR-3138	0.9433031	0.4352608
miR-4647	0.9406152	0.4347392
miR-652-3p	0.9351315	0.4329214
miR-4738-3p	0.9323295	0.4323412
miR-378e	0.9315274	0.4323412
miR-372-3p	0.9314976	0.4323412
miR-432-5p	0.9310364	0.4323412
miR-4648	0.9305149	0.4323412
miR-1284	0.9291692	0.4323412
miR-4327	0.9284499	0.4323412

Supplementary Table S4: List of 24 non-differentially expressed miRNAs using Alcohol as a main effect with their corresponding *p*-values and *q*-values.

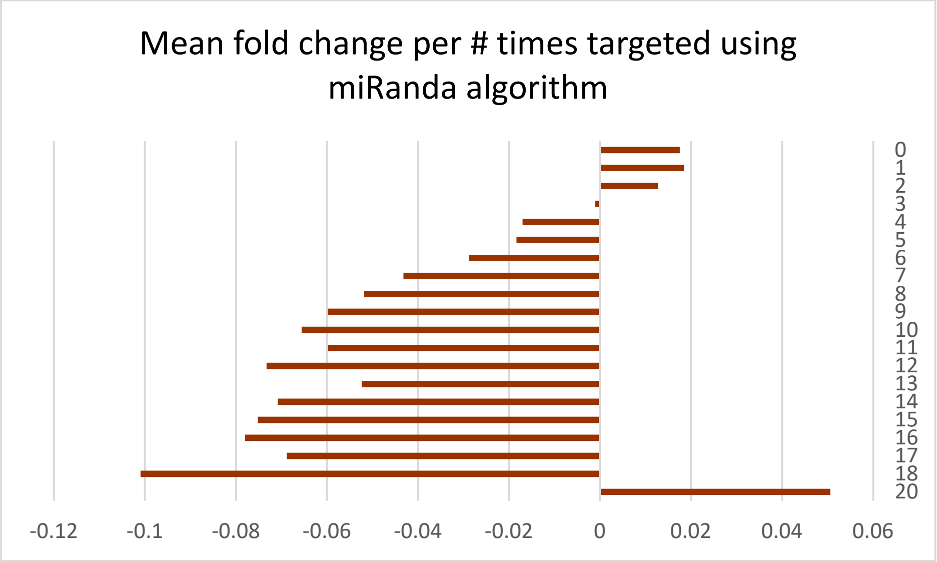

Supplemental Figure S6: Mean fold changes observed in our mRNA dataset using Alcohol as a main effect corresponding to the number of times they were independently targeted from a list of 22 upregulated miRNAs using the miRanda algorithm showing positive fold changes for the group of genes that had no predicted targets. The category of "20" corresponds to a single gene and likely represents a small sample size effect.
